# Life-History Traits of *Spodoptera frugiperda* Populations Exposed to Low-Dose Bt Maize

**DOI:** 10.1371/journal.pone.0156608

**Published:** 2016-05-31

**Authors:** Fernanda F. Sousa, Simone M. Mendes, Oscar F. Santos-Amaya, Octávio G. Araújo, Eugenio E. Oliveira, Eliseu J. G. Pereira

**Affiliations:** 1 Departamento de Entomologia, Universidade Federal de Viçosa, Viçosa, MG, 36570–900, Brazil; 2 Instituto Nacional de Ciência e Tecnologia em Interações Planta-Praga, Universidade Federal de Viçosa, Viçosa, MG, 36570–900, Brazil; 3 Núcleo de Fitossanidade, Embrapa Milho & Sorgo, Sete Lagoas, MG, 35701–970, Brazil; Pennsylvania State University, UNITED STATES

## Abstract

Exposure to *Bacillus thuringiensis* (Bt) toxins in low- and moderate-dose transgenic crops may induce sublethal effects and increase the rate of Bt resistance evolution, potentially compromising control efficacy against target pests. We tested this hypothesis using the fall armyworm *Spodoptera frugiperda*, a major polyphagous lepidopteran pest relatively tolerant to Bt notorious for evolving field-relevant resistance to single-gene Bt maize. Late-instar larvae were collected from Bt Cry1Ab and non-Bt maize fields in five locations in Brazil, and their offspring was compared for survival, development, and population growth in rearing environment without and with Cry1Ab throughout larval development. Larval survival on Cry1Ab maize leaves varied from 20 to 80% among the populations. Larvae reared on Cry1Ab maize had seven-day delay in development time in relation to control larvae, and such delay was shorter in offspring of armyworms from Cry1Ab maize. Population growth rates were 50–70% lower for insects continuously exposed to Cry1Ab maize relative to controls, showing the population-level effect of Cry1Ab, which varied among the populations and prior exposure to Cry1Ab maize in the field. In three out of five populations, armyworms derived from Bt maize reared on Cry1Ab maize showed higher larval weight, faster larval development and better reproductive performance than the armyworms derived from non-Bt maize, and one of these populations showed better performance on both Cry1Ab and control diets, indicating no fitness cost of the resistance trait. Altogether, these results indicate that offspring of armyworms that developed on field-grown, single-gene Bt Cry1Ab maize had reduced performance on Cry1Ab maize foliage in two populations studied, but in other three populations, these offspring had better overall performance on the Bt maize foliage than that of the armyworms from non-Bt maize fields, possibly because of Cry1Ab resistance alleles in these populations. Implications of these findings for resistance management of *S*. *frugiperda* in Bt crops are discussed.

## Introduction

For decades, sprays containing insecticidal proteins from *Bacillus thuringiensis* (Bt) have been used for pest management in agriculture, forestry, and public health [1]. The significance of Bt has increased dramatically with the introduction of transgenic crops producing Bt toxins to protect against major leaf, stem, and root feeding insect pests [2, 3]. Since 1996, Bt crops have been rapidly embraced by farmers worldwide [4]. Benefits of Bt crops include effective control of target pests, decreased use of conventional insecticides, reduced impact on non-target organisms, and increased farmer profitability [5–9]. However, the long-term efficacy of Bt toxins for pest management is threatened by evolution of resistance [10, 11]. Several studies have shown the high potential for Bt resistance evolution in laboratory and natural insect populations [12–16], especially in pest species adapted to warm climates, where rapid selection of resistant individuals [17, 18] can lead to field-relevant resistance [19, 20], characterized by reduced pest-control efficacy of the Bt technology against target insects.

The prevailing strategy proposed to manage resistance evolution by target pests in single-gene Bt crops involves a combination of a high dose of toxin produced in plant tissues and a refuge from exposure [10, 21]. The high dose must decrease the heritability of resistance by reducing its dominance, and the refuge must dilute the resistance alleles by promoting migration and mating of susceptible with resistant insects eventually emerging of the Bt crop. However, this strategy may not function properly if a sufficiently high concentration of toxin is not produced in the Bt plant such that a high proportion (>5%) of heterozygotes for Bt resistance survive exposure and transmit the resistance alleles to the next generation [10, 22]. Failure to meet the high-dose condition may be one of main circumstances that led to field-relevant resistance evolution in major pest species [19, 20, 23, 24].

Many Bt cultivars do not meet the high-dose criteria for the target pests [24], potentially generating sublethal toxin exposure in such pests. If a substantial proportion of Bt susceptible larvae recovers from sub-lethal intoxication on the Bt plant, and transmit susceptibility alleles to the next generation, they may contribute to slow down resistance evolution [10, 25]. However, low-dose Bt crops may also increase the risk of resistance evolution if the insecticidal protein titer in Bt cultivars kills most homozygous susceptible insects but allows for heterozygous insects (i.e., those carrying a single resistance allele) to pass the resistance allele to the next generation, increasing the rate of resistance evolution [26, 27]. This later scenario would be especially challenging where moderate-dose types of Bt crops are widely adopted without locally adapted, integrated insect resistance management [28].

Most Bt maize hybrids targeting the fall armyworm *Spodoptera frugiperda*, a major polyphagous lepidopteran pest in the western hemisphere, are not high-dose for the armyworm with one exception so far [29]. Low or moderate dose is especially true for transgenic maize events producing Cry1Ab (e.g., MON810, Bt11), a Bt toxin to which *S*. *frugiperda* larvae are relatively tolerant [30–32]. The relatively low susceptibility of fall armyworm to plants producing Cry1Ab gives us an opportunity to test whether sublethal exposure to concentrations of Bt toxins in maize plants may increase the rate of resistance evolution and compromise their efficacy against *S*. *frugiperda*, which was our aim in this study.

Here we compared life-history traits of five *S*. *frugiperda* populations collected from transgenic Bt or non-Bt maize and challenged with the Cry1Ab toxin in Bt maize foliage. We found that offspring of these *S*. *frugiperda* larvae previously exposed to Cry1Ab maize hybrids had variable fitness profile on Cry1Ab maize foliage in the populations studied, and three of them showed better performance on Cry1Ab maize leaves and no apparent fitness costs associated with the trait. Implications of these findings for resistance management of *S*. *frugiperda* in Bt crops are discussed.

## Materials and Methods

### Insect collection and rearing

**Fig 1:**
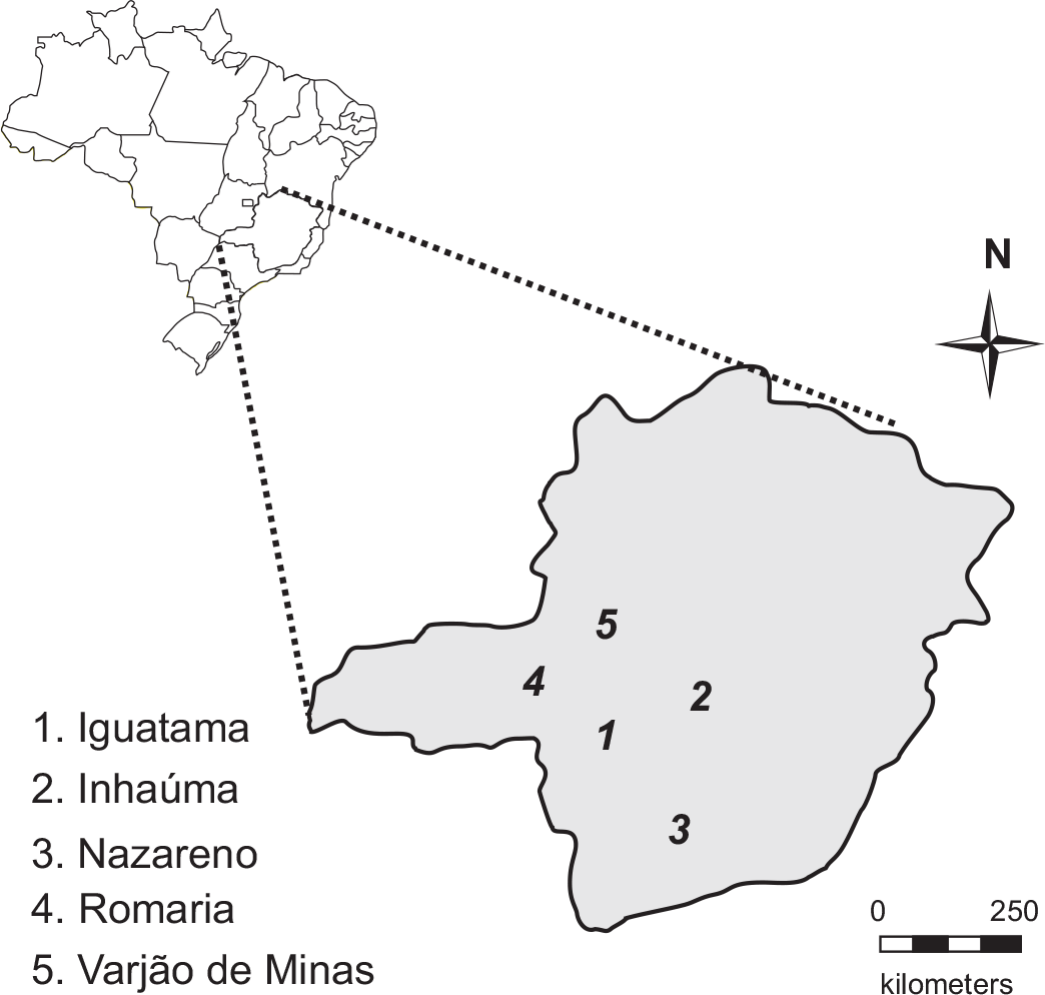
Sampling sites of the field populations of the fall armyworm, *Spodoptera frugiperda*. Shown is the graphical representation of Minas Gerais state, Brazil, with the locations of the counties where fall armyworms were collected. Eugenio E. Oliveira, co-author of this work, made the figure himself using a vector graphics editor. The authors are not aware of any previous copyrights on this figure, and it does not contain any proprietary data.

In the growing season of 2010, commercial fields of MON810 maize were identified by personnel from the Maize and Sorghum Center, Brazilian Agricultural Research Corporation (Embrapa Milho & Sorgo, Sete Lagoas, MG, Brazil) for field collections of *S*. *frugiperda* larvae. The collections were carried out on private lands (with the permission of their owners), and no specific permissions were required for these locations/activities as it did not involve endangered or protected species. All applicable international, national, and institutional guidelines for the care and use of the insects were considered in the present investigation. Bt Cry1Ab maize fields that had in their vicinity (within 1 km) comparable non-Bt hybrids in similar phenological stage were selected in five regions of high maize production from the State of Minas Gerais, Brazil (Fig 1). Most of the non-transgenic fields were refuge planted by local farmers. Late instar *S*. *frugiperda* larvae were collected by opening the whorl leaves containing typical armyworm injury and fresh larval frass. Field collections of 150–200 individuals were obtained from each site, and colonies were initiated with no fewer than 50 founder parents.

Field-collected larvae were placed on artificial diet in individual containers, coded according to location and host-plant type, and brought to the laboratory. Larvae were reared to adults on Bt-free artificial diet [33], and these were allowed to mate in cylindrical polyvinylchloride cages of 40 cm h x 30 cm dia. Moths were fed with a solution of 10% sugar and 5% ascorbic acid [33] and allowed to lay eggs on sulfite paper in the inner cage walls. Eggs were collected daily for three days during the oviposition peak and incubated in plastic bags with moistened filter paper until hatching. Neonates (F_1_) obtained from field-collected parents were maintained in the laboratory up to F_3_ using standard rearing techniques with artificial diet based on cooked dry beans, wheat germ, and casein [33]. For the F_1_-F_3_ rearing, neonates were placed on shredded diet and allowed to grow until third instar. Approximately 300 third-instar larvae were transferred to 50-ml translucent polystyrene cups (one larva/cup) with 5 ml of diet to minimize cannibalism. Pupation and adult emergence occurred within the cups. Emergent adults were transferred daily to mating cages and held as described previously. Insects were maintained in a rearing room at 27 ± 2°C, 70 ± 15% RH, and a photoperiod of 14:10 (L:D) h.

### Source of non-Bt and Bt maize leaf tissue

Two maize hybrids commercially available in Brazil were used for larval bioassays: Bt maize 30F35Y (event MON810, producing Cry1Ab) and its non-Bt isoline maize hybrid 30F35 (Dupont Pioneer, Santa do Cruz do Sul, RS, Brazil). Maize plants used were sown every two weeks in the experimental field of Embrapa Maize & Sorgo, Sete Lagoas, MG, Brazil. Plants were irrigated twice a day and fertilized on days 10 and 35 after emergence with 40 g of formulated 8-28-16 NPK fertilizer. The remaining crop management practices were applied according to the recommendations for the maize crop [34], without pesticide application and using mechanical weed control. Cry1Ab immunodetection assays using ImmunoStrip STX 06200/0050 (Agdia Inc., Elkhart, IN, USA) test strips were used according to the manufacturer's instructions to confirm the presence or absence of the Cry1Ab trait in the Bt or non-Bt isoline plants from which foliage were excised.

### Armyworm assays and experimental design

In the laboratory, we exposed fall armyworm larvae to foliage of Bt Cry1Ab maize and its non-Bt isoline. We used a factorial randomized experiment with two parental larval host plants (Bt Cry1Ab or non-Bt maize), five geographic locations of collection of the armyworms (see Fig 1), and two laboratory test plants or diet (Bt Cry1Ab maize or non-Bt isoline). The larvae were the F_3_ progeny of moths reared from the field-collected armyworms. Maize foliage was excised from whorl leaves of field-grown plants at V6-V9 stages [35], quickly placed in buckets with water, brought to the laboratory, thoroughly rinsed with distilled water, and placed on paper towels to dry for 15 min. The foliage was cut into 2-cm sections along the leaf blade and placed in 50-ml translucent plastic (i.e., polystyrene) to carry out the assays.

To set up the experiment, 48 batches of five neonates were assigned to control (i.e., non-Bt isoline) or Cry1Ab foliage. The sample size was 240 individuals assayed in four replicates or blocks of 12 cups (60 larvae) held in cup trays to facilitate handling. Using a fine hair brush, neonate larvae (< 24 h hatching) were placed in the 50-ml cups containing the excised leaf sections (5 neonates/cup). Cups were covered with plastic lids and held in the same environmental conditions described previously for insect rearing. We recorded 1^st^-instar survival rates after allowing the larvae to feed for 48 h. To record life-history traits up to the adult stage, a sample size of 72 survivors from the original cohorts of 240 larvae were placed singly in 50-ml cups and tracked throughout larval development under same conditions described previously. This design was replicated in three blocks (i.e., cup trays), each one comprising 24 cups with one larva per cup. Maize foliage was replaced every two days until pupation. Larval survival were recorded every two days, as were larval weight at 14 days, pupal weight 24 h after pupation, and development time from neonate to pupa. Survival rates on Bt maize foliage were adjusted based on natural mortality of larvae feeding on non-Bt maize (control) using Abbott's procedure [36].

### Estimating potential population growth rates

We investigated if the changes in life-history traits during larval development of *S*. *frugiperda* translate into differences in the potential for population growth in our experimental arrangement. Thus, in the adult stage we followed 10 pairs male-female or less depending on availability of adults because of treatment effects. Pupae from each treatment combination of parental host plant × location × test plant were separated by sex based on morphological differences of the last abdominal segments and held until adult emergence in 500-ml plastic cups lined internally with wet paper towel tissue. Ten pairs (replications) were formed for each combination of parental host plant × location × test plant by placing one pair male-female moths in a PVC cage (10 cm h x 10 cm dia.) and feeding them with a solution containing 10% sugar and 5% ascorbic acid. Cages were lined with sulfite paper sheets to provide oviposition substrate. The number of eggs masses laid by each female was recorded daily until the end of the oviposition period. Egg masses were individually transferred to 200-ml plastic cups, and the number of neonates hatched in each egg mass was recorded daily. The age and proportion of the cohort surviving to adult, as well as the sex ratio and number of females produced by each parental female were determined using the data recorded in the previous section as described elsewhere [37] with slight modifications. The intrinsic rate of population increase [38], or daily rate of female offspring production per parental female, (*r*_*m*_) was determined using the life-table format as described in following section.

### Statistical analysis

We used linear statistical modeling for data analysis of armyworm life-history traits, including survival, growth, and development. Normality and homogeneity of variances were checked for each response variable using residual analyses (PROC MIXED, PROC UNIVARIATE, PROC GPLOT) [39]. For larval survival rates at 48 h and at the end of the immature period, the data on the Cry1Ab test plants were adjusted for natural mortality on isoline non-Bt plants and subjected to a two-way analysis of variance (2 parental larval maize types × 5 populations or collection sites). There were four replications, each one comprising a tray with 12 cups of 50 ml with five neonates in each cup, totaling 240 larvae feeding on maize leaf sections in each treatment combination. For larval weight at 14 d, developmental time, and pupal weight, each tray containing 24 cups and 24 larvae (one larva/cup) was the replication (n = 3). These life-history traits were subjected to a three-way analysis of variance (2 parental larval maize types × 5 locations or collection sites × 2 laboratory test plants or diet types) and subsequently to mean separation using Fisher’s least significant difference procedure (PROC MIXED) [39] when appropriate.

Additionally, we used survival analysis to identify differences in the fall armyworm mortality schedule as affected by the factors under study: 5 locations of insect collection, 2 larval parental maize types, and 2 test plants (PROC LIFETEST) [39]. This a non-parametric procedure that uses Kaplan-Meyer estimators and yields chi-squared tests, as well as mean and median survival times for insects of each treatment combination and Tukey’s adjustment for multiple comparisons.

Finally, we estimated the intrinsic rate of population increase (r_m_, a statistic that summarizes information on immature development, reproduction, and survival) [38, 40] by interaction of the Lokta’s equation [41] using algorithms developed by Maia et al. [42] in SAS [39]. Pairwise comparisons for each combination of parental host plant × location × test plant were performed using one-tailed *t*-tests based on jackknife variance estimates [42].

## Results

**Fig 2:**
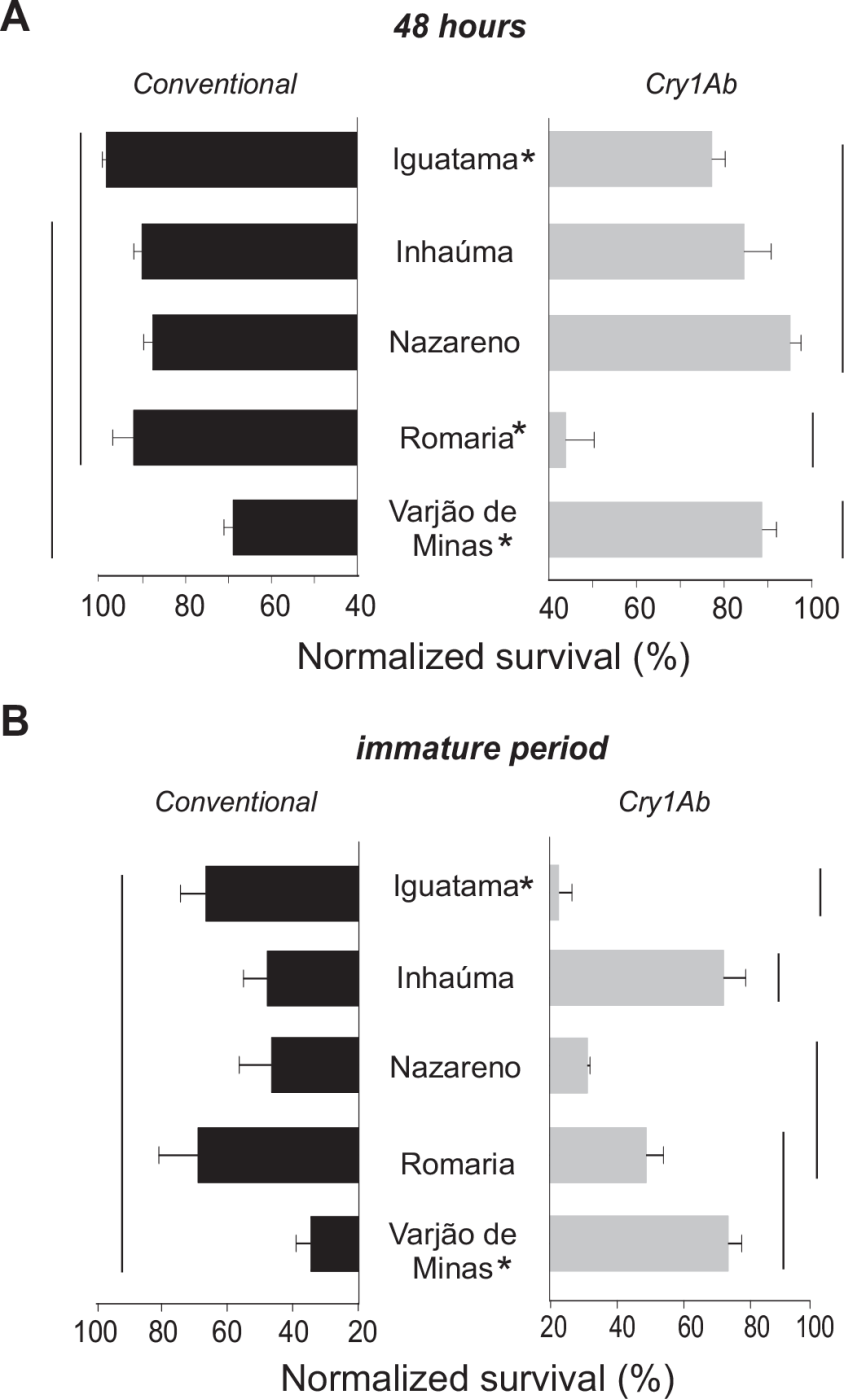
Survival rates for larvae of *Spodoptera frugiperda* from five populations chronically exposed to Cry1Ab throughout larval development. Insects were collected from conventional non-Bt (black bars) or Cry1Ab maize fields (grays bars) and their progeny reared on leaves of non-Bt isoline or Bt Cry1Ab maize in the laboratory. Survival on Cry1Ab maize foliage was adjusted (normalized) for natural mortality on non-Cry1Ab isoline (control) maize. A) Survival at 48h. B) Survival to adulthood. Means ± standard errors with the same line do not differ (*P* > 0.05) by Fisher’s protected Least Significant Difference procedure. Asterisk indicates significant difference (*P* < 0.05) between insects from Cry1Ab or non-Bt (conventional) maize fields.

Fall armyworm larval survival after 48 h (i.e., first-instar mortality) on Cry1Ab maize foliage varied among the site of insect collection (*F*_4, 30_ = 7.93, *P* < 0.001) and was affected by previous exposure to Cry1Ab maize (i.e., parental larval maize type) (*F*_1, 30_ = 10.33; *P* < 0.001) and by the interaction of these factors (*F*_4, 30_ = 15.58; *P* < 0.001), indicating that the first-instar armyworm survival on Cry1Ab maize foliage was variable with the field population and the recent past selection by the Bt maize in field. Armyworms from Bt maize (i.e., previously exposed to Cry1Ab) in Iguatama and Romaria produced offspring with fewer survivors than that produced from larvae collected in the non-Bt maize (i.e., without previous exposure the Bt toxin), indicating predominance of susceptible insects in these populations, while the opposite was observed for armyworms from Varjão de Minas, where larvae with prior exposure to Bt maize had significantly higher survival when reared on maize expressing Cry1Ab (Fig 2A).

Survival to adulthood on Cry1Ab maize (adjusted for natural mortality on non-Bt maize) depended on the interaction collection site × parental larval maize type (*F*_4, 20_ = 6.59, *P* < 0.01), but no main effect of collection site or prior exposure to Cry1Ab maize was observed (*P* > 0.05). Across the collection sites and parental larval host plant, a wide variation in armyworm survival rates was observed, ranging from 22.8 ± 3.9 to 73.6 ± 4.0% (mean ± SE). For armyworms from Varjão de Minas, offspring of larvae with prior exposure to Cry1Ab maize had increased survival rates in relation to those without prior exposure, indicating that these individuals may carry Cry1Ab resistance alleles. Conversely, for insects of Iguatama, offspring of larvae collected on Cry1Ab maize survived significantly less than those collected on non-Bt maize (Fig 2B), indicating that these individuals were significantly more susceptible to Cry1Ab than individuals of the other locations.

**Fig 3:**
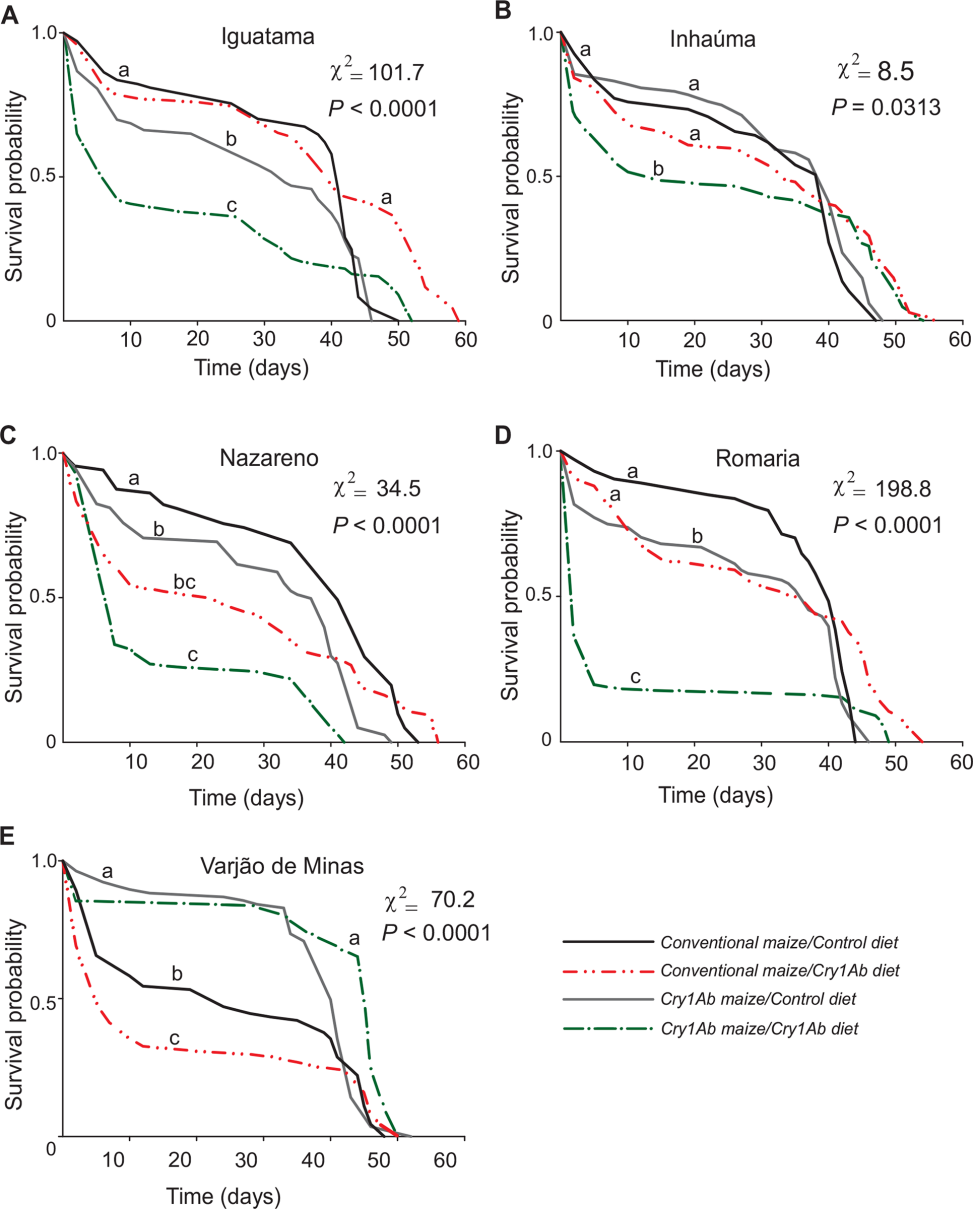
Survival plots of five populations of *Spodoptera frugiperda* chronically exposed to Bt Cry1Ab maize throughout larval development. Insects were collected from conventional (non-Bt) or Cry1Ab maize fields and their progeny reared on leaves of non-Bt maize (i.e., control diet) or Bt Cry1Ab maize (i.e., Cry1Ab diet) in the laboratory. Survival curves that do not significantly differ (*P* > 0.05) were coded with the same letter.

The mortality schedule for armyworms of each collection site as affected by prior exposure to Cry1Ab maize and by continuous exposure to this Bt toxin throughout larval development is shown in Fig 3. As expected, the hypothesis that all survival curves were similar in all 20 treatment combinations was rejected (*χ*^*2*^_19_ = 489.12, *P* < 0.001). For each one of the five collection sites (i.e., Iguatama, Inhaúma, Nazareno, Romaria, and Varjão de Minas), the armyworm mortality schedule was significantly different between cohorts with and without prior recent exposure to Cry1Ab maize (i.e., whether or not collected on Cry1Ab maize) as well as between cohorts reared on non-Bt maize foliage (i.e., control diet) and exposed to Cry1Ab maize foliage throughout larval development (*P* < 0.05) (Fig 3). Insects from Nazareno and Romaria were the most susceptible ones to chronic exposure to Cry1Ab maize in the laboratory as their larvae died faster than those reared on control maize diet. Conversely, the Varjão de Minas population collected in Cry1Ab maize field was the least susceptible one to the Bt maize as its survival curve was similar when fed non-Bt isoline or Cry1Ab maize leaves.

**Fig 4:**
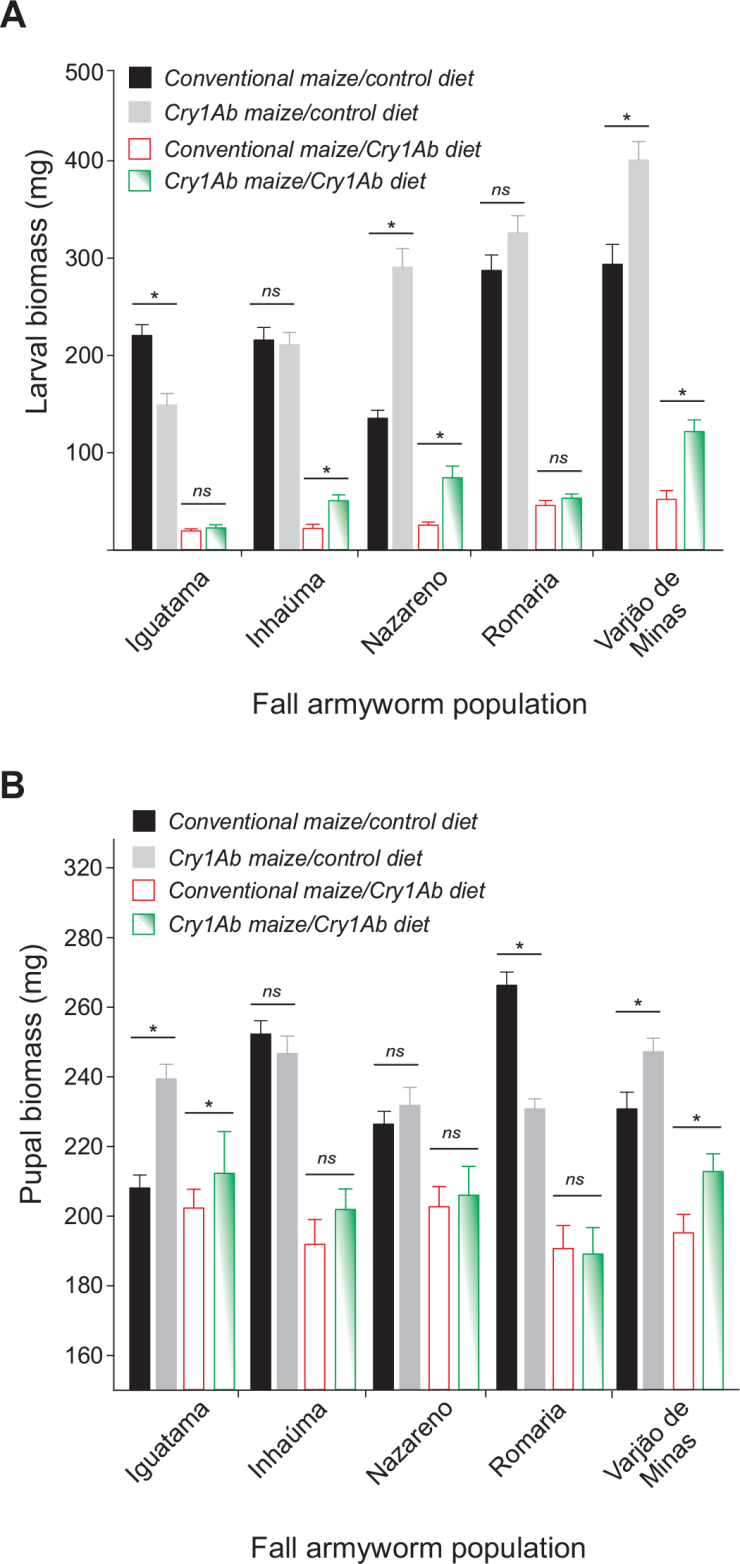
Body size of *Spodoptera frugiperda* from five populations chronically exposed Bt Cry1Ab maize throughout larval development. Insects were collected from conventional (non-Bt) or Cry1Ab maize fields and their progeny were reared on leaves of isoline or Cry1Ab maize in the laboratory. A) Larval weight gain 14 days after hatching. B) Pupal weight 24 h after pupation. While means ± standard error with asterisk differ significantly (*P* < 0.05, Fisher’s protected Least Significant Difference procedure) between insects of the same population reared on non-Bt (i.e., control diet) or Bt Cry1Ab maize foliage, means ± standard error with **ns** indicate no significant difference.

Cry1Ab maize foliage reduced armyworm larval weight gain in relation to non-Bt maize foliage (*F*_1, 40_ = 1182, *P* < 0.001) regardless of the larval parental host plant and location of collection sites (Fig 4A). Larvae reared on non-Bt maize foliage weighed (mean ± SE) 257.3 ± 16.2 mg while those reared on Cry1Ab maize weighed 48.3 ± 5.8 mg (Fig 4A), which correspond to (mean ± SE) 81.2 ± 2.0% larval growth inhibition (see S1 Fig for growth inhibition of all *S*. *frugiperda* populations). Interestingly, fall armyworm larvae seem to have compensated the sublethal effect of Cry1Ab maize on growth inhibition as the magnitude of the reduction in the pupal weight on Cry1Ab foliage in relation to non-Bt foliage (i.e., control diet) was smaller than that observed in the larval growth inhibition data (Fig 4A and 4B).

**Fig 5:**
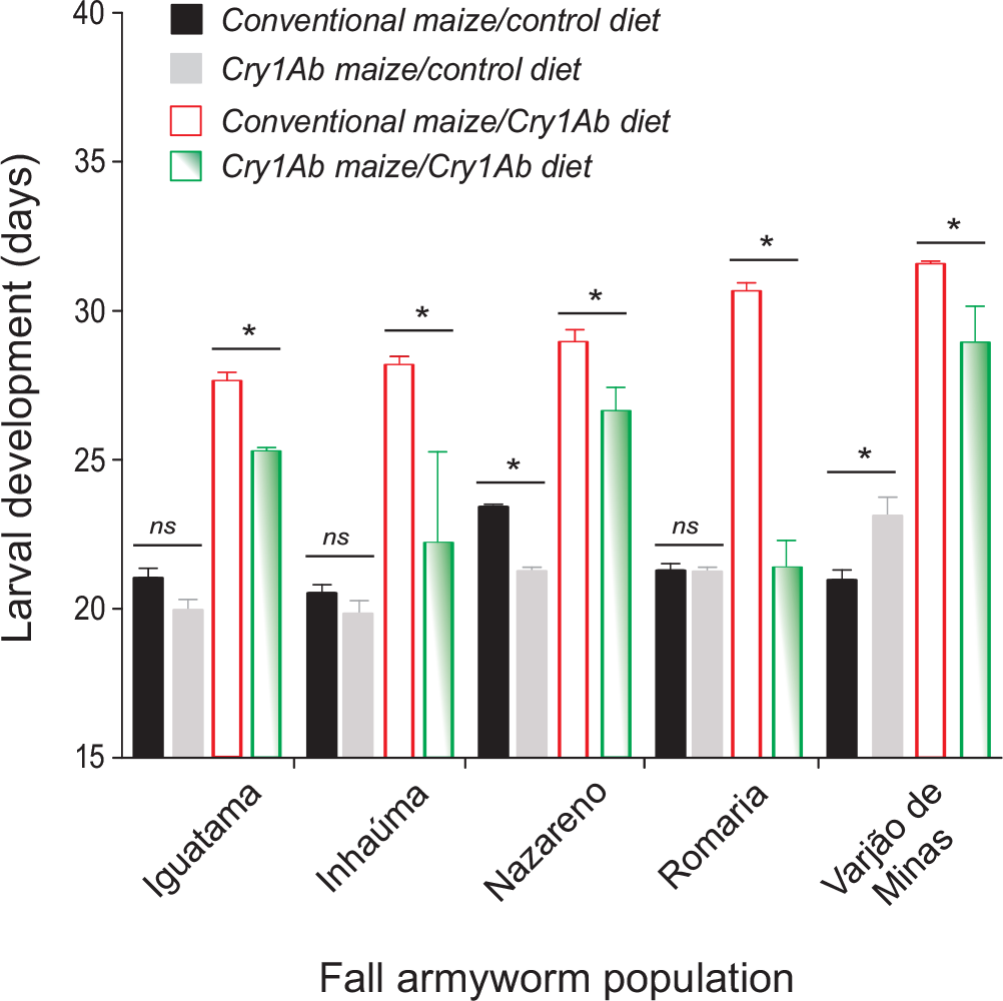
Increase in the developmental time of five *Spodoptera frugiperda* populations caused by continuous exposure to Bt Cry1Ab maize foliage throughout larval development. Data are mean time to develop from neonate to pupa for five populations of fall armyworm collected from conventional (non-Bt) or Cry1Ab maize fields and reared on leaves of non-Bt isoline (i.e., control diet) or Bt Cry1Ab maize (i.e., Cry1Ab diet) in the laboratory. While means ± standard errors with asterisk significantly differ (*P* < 0.05, Fisher’s protected Least Significant Difference procedure) between insects of the same population fed non-Bt isoline or Cry1Ab maize leaves as diet, means ± standard errors with ns indicate no significant difference.

Likewise, continuous exposure to Cry1Ab maize leaves was a major factor prolonging larval development time (*F*_1, 40_ = 1007, *P* < 0.001) regardless of the parental larval host plant or the location of collection (Fig 5). Armyworms reared on non-Bt maize foliage took (mean ± SE) 21.3 ± 2.5 days to develop from neonate to pupa while those reared on Cry1Ab maize foliage took 28.5 ± 3.9 days to complete larval development; thus, Cry1Ab toxin in maize leaves consistently caused a 7-day delay in fall armyworm larval development time. Importantly, such developmental delay (i.e., sublethal effect) caused by Cry1Ab was lower on larvae derived from armyworms collected in Cry1Ab maize fields, indicating that if the parental larvae is prescreened on this Bt maize and maintained in a closed population (i.e., without immigration of susceptible individuals), Cry1Ab resistance alleles can be passed on the offspring and may build up at least in some populations.

**Fig 6:**
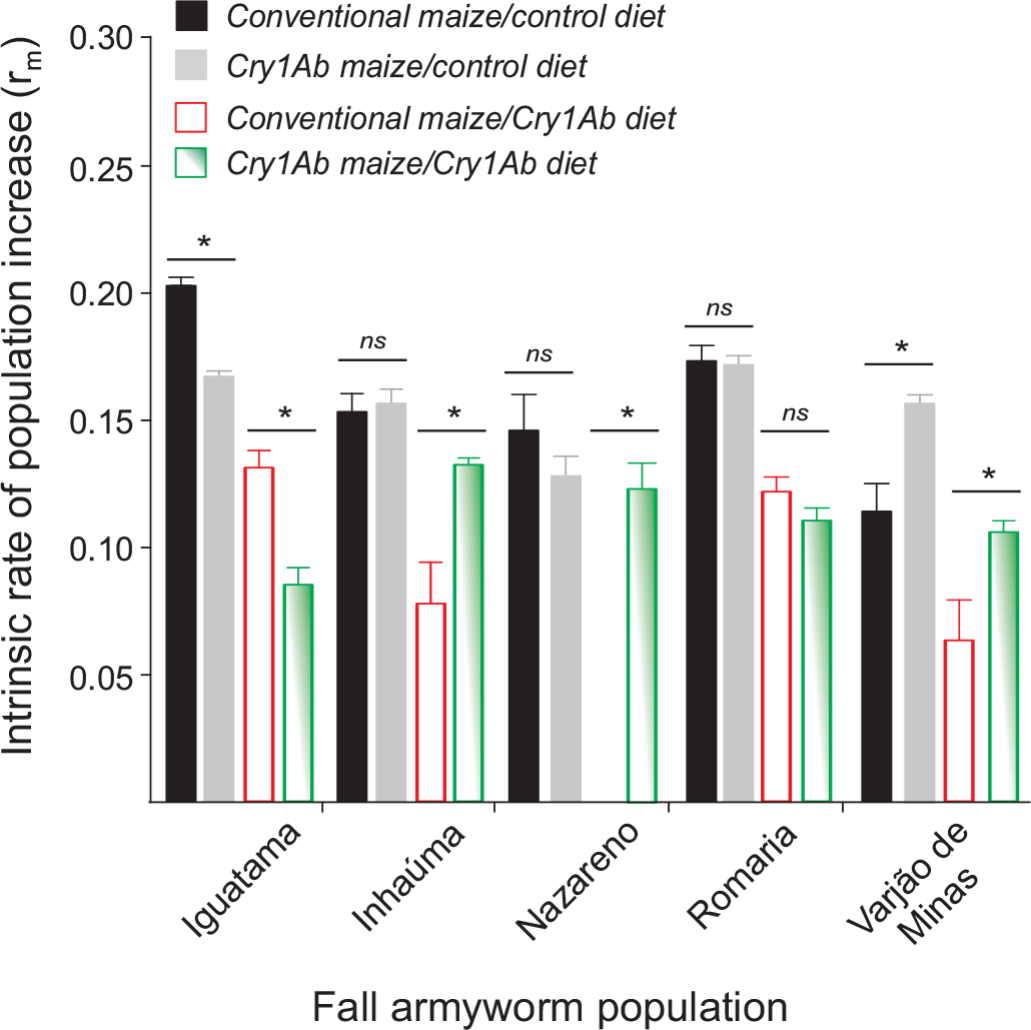
Fitness index for five *Spodoptera frugiperda* populations exposed to Bt Cry1Ab maize. Insects were collected from conventional non-Bt or Cry1Ab maize fields and their progeny were reared on leaves of non-Bt isoline (i.e. control diet) or Cry1Ab maize foliage (i.e., Cry1Ab diet) in the laboratory. Data are estimates of intrinsic rate of population growth obtained using the life table format, and error bars are 95% confidence intervals. While asterisk indicates significant difference (*P* < 0.05) by one-tailed *t*-test using variances estimated by the jackknife technique in SAS [42], **ns** indicate no significant difference.

Intrinsic rates of population increase (r_m_) calculated for armyworm cohorts reared on control or Cry1Ab maize foliage indicated complex interactions in the potential population growth for armyworms with or without prior exposure to Cry1Ab maize (Fig 6). Population growth rates were 50–70% lower for individuals continuously exposed to Cry1Ab maize relative to controls; for insects of Nazareno obtained in non-Bt maize, r_m_ values could not even be estimated as the cohort did not reproduce (and had reduced larval survival, growth, and development, Figs 2, 4 and 5), hence showing the population-level effects of Cry1Ab exposure in *S*. *frugiperda*, which varied with population source and their prior exposure to Cry1Ab maize in the field. In some populations (e.g., Iguatama, Romaria), r_m_ values for insects collected in Cry1Ab maize were lower on Cry1Ab maize test foliage than on non-Bt maize foliage (i.e., control diet), indicating that Cry1Ab susceptibility alleles prevailed in the offspring of insects prescreened with this Bt toxin in the field (Fig 6). For the Iguatama population, a fitness cost of the past selection on Cry1Ab maize was evident in the lower r_m_ values for insects collected in Cry1Ab maize and reared on non-Bt maize diet.

Importantly, on Cry1Ab maize foliage, armyworms of three populations (Inhaúma, Nazareno, and especially Varjão de Minas) collected on Cry1Ab maize showed higher r_m_ values than those collected on non-Bt maize (Fig 6), which is consistent with their better performance obtained for other life-history traits in the presence of the Bt toxin (Figs 3–5). These populations had no fitness disadvantages of the Cry1Ab-tolerance trait in the absence of Bt protein (i.e., non-Bt maize foliage or ‘control diet’) as indicated by their similar or higher r_m_ values in relation to insects that the parental larval host plant was non-Bt maize (Fig 6). Interestingly, for the Varjão de Minas population, r_m_ values (i.e., fitness) for insects collected on Bt maize was even higher than those obtained for insects collected on non-Bt maize in same location regardless of the rearing environment; hence, their increased performance on Cry1Ab maize (i.e., inherited increased tolerance) clearly does not impose a fitness cost on the population growth potential and apparently confer a fitness advantage.

## Discussion

Although sublethal effects of *B*. *thuringiensis* toxins on target insects of transgenic crops have been recognized as important to interpret their population-level effects [43–47], studies integrating both lethal and sublethal effects of Bt toxins on *S*. *frugiperda* life history are scarce to date. This species is a highly mobile insect pest in a wide range of host crops in the Neotropical America [48], where it is one of the main targets of the Bt transgenic technology and is notorious for holding two of the six cases of field-relevant resistance evolution to Bt Cry1F maize [19, 20, 24], for field-evolving resistance to Cry1Ab [32], and for having high potential to adapt to dual-gene Bt maize producing Cry1A.105 + Cry2Ab [17]. Here, we show that continuous exposure of immature stages of fall armyworm to Cry1Ab maize foliage containing transformation event MON810 affects the rates of larval survival, growth and development, as well as pupal weight and the intrinsic rate of population increase in various field-derived populations, and to some extent, these effects depended on the host plant (Cry1Ab or non-Bt maize) the parental larvae were exposed to.

Cry1Ab maize caused lethal and sublethal effects on fall armyworms as indicated by the variable rates of 75–95% growth inhibition and the 20–80% reduction of larval survival in the populations studied (see Figs 2–4, and S1 Fig). These levels of mortality and growth inhibition are in agreement with other studies on fall armyworm from Brazil [32, 49] and other pests that are relatively tolerant to Bt toxins [26, 27] or on Bt cultivars of variable Cry1Ab titer [50]. These best levels of mortality and growth inhibition for fall armyworm clearly show that MON810 maize hybrids do not meet the high-dose condition for *S*. *frugiperda* (i.e., > 99.9% larval mortality) [51], which should be considered for proper resistance management of fall armyworm to Cry1Ab maize [52–54].

For some collection sites (e.g., Iguatama and Romaria), armyworms populations obtained in Cry1Ab maize (i.e., with recent past selection by the Bt toxin) produced offspring with fewer survivors than those produced from larvae collected in non-Bt maize fields (i.e., without recent past selection by Cry1Ab) (see Figs 2 and 3), and this was consistent with their poorer performance profile in the other life-history traits (i.e., lower weight gain and population growth potential, Figs 4 and 6). These findings indicate that Cry1Ab susceptible larvae recovered from sub-lethal intoxication on the Bt maize and transmitted susceptibility alleles to subsequent generations, which may have contributed to slow rates of resistance evolution to Cry1Ab [32] as compared to the relatively rapid resistance evolution to Cry1F in fall armyworm field populations [19, 20, 55]. In addition, lower adoption rates of Cry1Ab maize by farmers because of the availability of other Bt maize hybrids with higher control efficacy against *S*. *frugiperda* in the country [56, 57] must have contributed to lessened selection pressure for Cry1Ab resistance evolution, and this technology can still be valuable in an integrated pest management approach using multiple control measures to reduce pest population density.

Lethal and sublethal effects were observed in all fall armyworm populations regardless of prior exposure, population source, and rearing environment. Even so, we found evidence of some fitness gain associated with previous exposure to Cry1Ab maize in three of five collection sites (Inhaúma, Nazareno, and Varjão de Minas). In these locations, the armyworm colonies collected on Cry1Ab maize had higher intrinsic rate of population increase when challenged with Cry1Ab maize foliage in relation those which the parental larvae were from non-Bt maize. Interestingly, these apparently higher levels of Cry1Ab resistance in insects collected in Cry1Ab maize do not seem to carry a cost for the armyworms as they had no reduced fitness on the non-Bt maize foliage. In the other spectrum of the variation was the Iguatama population collected from Cry1Ab maize fields, which showed a poorer fitness profile for survival, weight gain, and population growth rate on non-Bt maize foliage (see Figs 2–4 and 6), indicating a fitness cost of the previous exposure to Cry1Ab maize in the field. Fitness costs favor natural selection against resistance, but their lack thereof may allow for directional selection of Cry1Ab resistant individuals [58] in at least some populations.

As clearly demonstrated here for fall armyworm on Cry1Ab maize, Bt toxin exposure in some Bt crops can prolong larval development. Interestingly, larval development time was reduced in individuals prescreened with Cry1Ab in the field (see Fig 5), which is similar to a pattern observed in the laboratory with Cry1F [59] and Cry1A.105 + Cry2Ab [17]. These findings indicates the delayed development caused by Bt toxins in fall armyworm affects mainly susceptible or partially resistant individuals but not so much completely resistant ones. Delays in larval development time on Bt crops may either increase or decrease the rate of resistance evolution, depending on complex interactions. First, if Bt-exposed individuals have delayed development time that desynchronizes their maturation in relation to those feeding on non-Bt plants (i.e., refuge), it may favor assortative mating between resistant individuals, compromising refuge deployment [10, 60]. However, in field settings this issue may be diminished as *S*. *frugiperda* as has multiple and overlapping generations year around, which may produce susceptible moths for mating with Bt resistant ones, provided that Bt resistance, larval development in different host plants, or other sublethal effects of Bt toxins do not disrupt normal pheromone communication or the reproductive behavior of the moths.

In addition, extended larval stages tend to increase the likelihood of mortality by natural enemies [10, 61–64], which can delay resistance development to Bt plants when maintaining a low pest density and low crop damage [65]. In fact, damage by fall armyworm seem to be minimized when Bt maize is concurrently used with biological control [66, 67], especially small predatory bugs, which are found abundantly in maize fields and preys preferentially on small fall armyworm larvae stunted by sublethal intoxication on Cry1Ab maize [68]. Furthermore, in four out of five locations fall armyworm larvae challenged on Cry1Ab maize foliage had reduced rate of population growth (see Fig 6), such that a reduced number of individuals may be passing resistance alleles to the next generation, thus slowing resistance evolution at least some field populations. Altogether, these findings and observations help explain why field performance of Cry1Ab-producing maize hybrids against *S*. *frugiperda* in Brazil have not reduced much since the beginning of their commercialization [56, 57, 67, 69, 70] despite evolution of field resistance in some populations [32]. In addition to *S*. *frugiperda*, Cry1Ab maize help reduce population density of other lepidopteran species, such as *Diatraea saccharallis*, *Elasmopalpus lignosellus*, and *Helicoverpa* spp., thus having value for integrated pest management of multiple pests in the Brazilian agricultural landscape, particularly using multiple approaches to reduce pest pressure levels and judicious interventions with chemical applications to delay resistance development.

We observed a wide variation in fall armyworm to response Cry1Ab maize foliage (i.e. variable rates of survival, weight gain, and population growth), and in part our findings agree with those published recently [32], showing a 12-fold variation in Cry1Ab susceptibility in 2000/2001 surveys and a mean reduction in larval growth inhibition from 2010–2015 at a diagnostic concentration with increased variation in the response of fall armyworm populations to the toxin. Such variation in Cry1Ab susceptibility is not unexpected based on our data on developmental delay and a modeling study [71], which found that delays in larval development time on Bt crops may either increase or decrease the rate of resistance evolution, depending on complex interactions as discussed above.

Apart from implications for resistance management, we identified a range of sublethal effects when fall armyworm larvae were exposed to a low-dose Bt maize producing Cry1Ab, and more research is needed to better understand its overall ecological impact and interpret insecticidal protein efficacy in controlling target insect populations [47]. Also, whether or not exposure to subtethal exposure to Bt toxins induce hormesis-like responses [72] potentially will help us to update information important to devise and refine resistance management strategies for low or moderate-dose transgenic plants used against Bt tolerant lepidopteran species [15, 57, 73–75]. Our data suggest that some Bt susceptible larvae recovered from sub-lethal intoxication on low-dose Bt plants and transmitted susceptibility alleles to subsequent generations, and thus low or moderate expression of Bt toxin genes in plants combined with effective refuge may delay resistance development, especially when fitness costs of surviving sublethal intoxication helps to hinder the increase of resistance alleles in the exposed insect population.

In summary, transgenic Bt maize producing Cry1Ab had a range of lethal and sublethal effects on *S*. *frugiperda* populations regardless of their prior larval development on the transgenic maize and geographic location of the armyworm collections. Three out of the five populations derived from parental larvae surviving field-exposure to Cry1Ab maize had increased demographic performance on Cry1Ab maize foliage (i.e., they inherited resistance alleles) and no reduced fitness on non-Bt maize (i.e., the resistance trait carried no cost); the other two populations collected in Cry1Ab maize fields had reduced fitness on Cry1Ab maize foliage (i.e., they inherited susceptibility alleles) and one population had reduced fitness non-Bt maize (i.e., fitness costs of past exposure to Cry1Ab maize), indicating that prior exposure of fall armyworm to Bt Cry1Ab maize is not consistently correlated with increased tolerance to this type of Bt maize in subsequent generations. We found evidence that some Bt susceptible fall armyworm larvae recovered from sub-lethal intoxication on Cry1Ab plants and transmitted susceptibility alleles to subsequent generations, and this feature help explain the relatively slow increase of Cry1Ab resistance alleles in field populations of fall armyworm. The field-derived populations of *S*. *frugiperda* with increased fitness on Cry1Ab maize provide opportunity to investigate the genetics/molecular basis of Cry1Ab resistance and update information that will worthy to refine resistance management strategies for lepidopteran pest species to which is difficult to obtain high-dose Bt transgenic events.

## Supporting Information

S1 Fig Growth inhibition of *Spodoptera frugiperda* from five populations chronically exposed Bt Cry1Ab maize throughout larval development. Insects were collected from conventional non-Bt (black bars) or Cry1Ab maize fields (grays bars) and their progeny reared on leaves of non-Bt isoline or Bt Cry1Ab maize in the laboratory. Survival on Cry1Ab maize foliage was adjusted (normalized) for natural mortality on non-Cry1Ab isoline (control) maize. Means ± standard errors with the same line do not differ (*P* > 0.05) by Fisher’s protected Least Significant Difference procedure. Asterisk indicates significant difference (*P* < 0.05) between insects from Cry1Ab or non-Bt (conventional) maize fields.
(EPS)
